# *In vivo* imaging of patients with chronic pruritus of unknown origin reveals partial sweat duct obstruction with partial itch resolution upon retinoid treatment

**DOI:** 10.3389/fmed.2023.1265148

**Published:** 2023-09-22

**Authors:** Shi Yu Derek Lim, Yingrou Tan, Yuning Zhang, Xiahong Zhao, Lai Guan Ng, Hong Liang Tey

**Affiliations:** ^1^National Skin Centre, Singapore, Singapore; ^2^Singapore Immunology Network, Agency of Science and Technology, Singapore, Singapore; ^3^Lee Kong Chian School of Medicine, Nanyang Technological University, Singapore, Singapore; ^4^Yong Loo Lin School of Medicine, National University of Singapore, Singapore, Singapore

**Keywords:** acitretin, dermcidin, imaging, isotretinoin, pruritus, retinoids, sweat

## Abstract

**Background:**

Chronic pruritus of unknown origin (CPUO) is poorly understood and lacks effective treatment options.

**Objectives:**

We aimed to elucidate abnormalities in the sweat apparatus of patients with CPUO, and to assess efficacy and safety of treatment with systemic retinoids.

**Methods:**

An initial case–control study included 20 affected patients and five healthy controls, for whom heat and sweating were induced, either through a standardized exercise protocol or ingestion of hot water. *In vivo* high-definition optical coherence tomography, whole-body starch-iodine testing, and skin biopsy for immunofluorescence staining were done to evaluate for sweat duct obstruction. A subsequent retrospective cohort analysis included 56 patients with CPUO, seen at an Itch subspecialty clinic of a single tertiary referral centre, who failed conventional treatments and were treated with isotretinoin and/or acitretin from May 2014 to November 2020. Treatment response to retinoids was defined as a sustained reduction in itch score of ≥2/10. Safety was assessed by proportion stopping treatment due to side effects.

**Results:**

*In vivo* imaging in 19 (95%) patients revealed features of partial keratinaceous sweat duct obstruction with statistically significant luminal dilatation compared to controls. Immunofluorescence studies of three patients’ paired lesional/non-lesional biopsies revealed dermcidin accumulation within sweat glands coupled with dermcidin leakage in itchy skin. Fifty-six patients (mean [SD] age 55.2 [17.5] years, 69.6% male) were treated with systemic retinoids. Mean (SD) duration of itch was 116.3 (140.4) months and mean (SD) itch score was 8.2 (1.8). Forty-one (73.2%) initially received isotretinoin, and 15 (26.8%) acitretin. At three months, mean itch score reduced by 2.38 (95% CI -3.2 to −1.6, *p* < 0.0001). Thirty-eight (67.9%) had a sustained response. Eight (14.81%) achieved an itch score of 0 or 1, with four stopping treatment for a mean (SD) of 318.5 (291.2) days without relapse. Eight (14.3%) stopped or switched retinoid due to adverse effects, with similar incidences between both retinoids, the commonest being dryness.

**Conclusion:**

Based on novel findings from physiological imaging studies identifying partial keratinaceous sweat duct obstruction in CPUO, we instituted systemic retinoid treatment to address the underlying pathology. In patients who failed conventional therapies, the treatment appears effective and safe.

## Introduction

The point prevalence of chronic pruritus, defined as itch lasting for longer than six weeks, is estimated to be 13.5% in the general adult population, with the highest prevalence amongst the elderly (1). In 8–15% of affected patients, no causation or known associations are found, and these patients are diagnosed to have chronic pruritus of unknown origin (CPUO) (2). The pathogenesis of CPUO is poorly understood and there is a paucity of effective treatment options. A systematic review revealed an absence of evidence for efficacious interventions, such as topical emollients, cooling lotions, corticosteroids, antidepressants, antihistamines, anticonvulsants, and phototherapy (3). The medical and psychosocial morbidity of this disease cannot be over-emphasized, with 18.5% of patients reporting suicidal ideations due to pruritus in the course of their disease (2). Thus, it is imperative to identify the key pathogenic mechanisms and to uncover effective treatments for CPUO.

In our previous study on isolated hypohidrosis, where patients are unable to sweat on more than 40% of the body surface area, we found the presence of hypo-refractile material below the stratum corneum together with dilated sweat ducts that were not present in healthy individuals using high-definition optical coherence tomography (HD-OCT) (4). These findings suggested that the sweat ducts were obstructed with consequent pooling of sweat in the epidermis. Subsequent histological analysis of patients’ skin biopsies revealed keratinaceous deposits in the superficial sweat ducts. More than 85% of these patients responded to treatment with systemic retinoids and remained well long term after stopping retinoid treatment, which was directed toward resolving the keratinaceous obstruction at the sweat orifices. Interestingly, more than 40% of these patients experienced itch, suggesting that itch could be associated with sweat orifical obstruction. Retinoids are known to reduce hyperkeratosis accounting for sweat duct obstruction, and have been demonstrated to reverse hypohidrosis and improve thermoregulation in congenital ichthyoses (5). This provides the scientific basis upon our investigation into the use of retinoids to resolve sweat duct blockage in itch.

Numerous previous studies have found the association between itch and sweat duct blockage. In 1947, Sulzberger published a finding of a blocked acrosyringium in a patient suffering from atopic dermatitis (AD), postulating that the blockage of the sweat duct is associated with the AD disease process (6). Similarly, parakeratotic plugging of the sweat duct was observed in lesional AD skin, with ductal distension following thermal initiation of sweating. More recently, Haque et al. and Allen et al. showed sweat duct blockage was caused by biofilm forming bacteria *Staphylococcus epidermidis* and is linked to miliaria, with 100% of 36 AD skin samples having blocked sweat ducts containing bacteria, together with associated inflammatory infiltrates (7, 8). Indeed, a commonly reported trigger of itch is the exposure to heat or initiation of sweating, during which there is stimulation of the sympathetic nervous system (9). Together, these studies suggest that obstruction of the acrosyringia can result in subclinical miliaria, inciting itching and scratching (8).

In our preliminary studies with non-invasive *in vivo* imaging using high-definition optical coherence tomography (HD-OCT) of individuals with CPUO, similar features of sweat duct obstruction were observed, akin to those observed in our patients suffering from hypohidrosis (4). We were therefore interested to study the relation between sweat duct abnormalities with reduction of sweat duct blockage using systemic retinoids. Hence, we aimed to elucidate abnormalities in the sweat apparatus of patients with CPUO using HD-OCT and immunofluorescence studies in biopsies from such patients and to investigate the efficacy and safety of treatment in CPUO patients using systemic retinoids to reduce sweat duct blockage in a retrospective cohort analysis. Supplementary Figure S1 depicts the respective studies performed on the subjects in our study.

## Materials and methods

### Clinical *in vivo* imaging

Patients with CPUO first underwent baseline noninvasive *in vivo* skin imaging using HD-OCT (Skintell®; Agfa, Belgium). Each image volume captures a 1.8 × 1.5 mm area of skin to a depth of 0.5 mm, in which slice and *en face* images can be simultaneously visualized, allowing for 3-dimensional assessment of the skin. Heat and sweat induction were subsequently performed by getting patients to exercise on a stationary bicycle for 20 min in a temperature- (30–33°C) and humidity-regulated (65–75%) room. For subjects who were unable to perform the exercise, heat stimulation was performed by asking them to drink hot water in a warm room (28–30°C), until they started sweating on any part of their body.

Subsequently, all the patients underwent repeat *in vivo* skin imaging using HD-OCT (Skintell®; Agfa, Belgium). These images were compared to HD-OCT skin images obtained from five healthy volunteers obtained at baseline and post-exercise. Four to six measurements of sweat duct lumen diameter and wall thickness were obtained for all participants, with the mean values for each subject recorded. One-way analysis of variance was used to assess for statistical differences in the means of the different patient groups.

Patients who could perform the cycling exercise also underwent a whole-body starch-iodine test, as described in our previous publication (10). This test is able to accurately identify if individuals are able to sweat on all skin areas. The presence of hypohidrosis is defined by the inability to sweat affecting >40% of body surface area, as previously described (11).

Three of the CPUO patients underwent two skin biopsies each, one obtained from an itchy region and another from an adjacent non-itchy region. Skin biopsies were cryosectioned and processed for indirect immunofluorescence staining with mouse monoclonal antibody (G-81) labeling for dermcidin (Santa Cruz), an anti-microbial peptide produced in sweat glands and imaged using the EVOS slide scanner (Thermo Scientific) at 20x magnification.

### Retrospective study on retinoid treatment

In relation to our preliminary findings, we had routinely instituted treatment of CPUO using systemic retinoids for patients who have failed conventional therapies, in the attempt to regulate abnormalities in keratinization at the skin surface (12, 13). We performed a retrospective cohort analysis of all patients with a diagnosis of CPUO, managed in our subspecialty Itch Clinic, treated with systemic retinoids over a 6.5-year period between 16 May 2014 and 6 November 2020. Twenty of these patients had undergone *in vivo* skin imaging, as described in the section above, based on their willingness to undergo additional investigations. We included only patients with at least 6 weeks of itch and no clinically evident primary dermatological abnormalities (except for scratch-induced skin lesions). All patients had failed treatment with topical emollients and steroids, and at least three types of antihistamines from different generations at three to four times the recommended daily dosage. The patients had been treated with isotretinoin and/or acitretin off-label for their itch. We excluded patients who were unable to provide a numerical itch score, those diagnosed with a primary dermatosis or systemic disease accounting for their pruritus, and those who did not return for at least one follow-up visit after starting systemic retinoids.

Patient demographics, disease characteristics, verbalization of suicidal ideation, retinoid treatment doses and duration, and adverse effects due to itch were collected. To assess the efficacy of retinoid treatment, we evaluated the itch score of each patient at each follow-up visit. The itch score reflects the patient’s average intensity of itch over the prior 3 days on a 0–10 numerical rating scale. We defined treatment response as a reduction in itch score of two points or more, which is sustained during the treatment course. The itch score value of two was selected in accordance with the previously determined Minimal Clinically Important Difference in numerical rating of chronic itch (14). Safety was assessed by the number of patients who stopped either or both retinoids due to side effects.

All variables were summarized descriptively using counts and percentages for categorical variables and mean with standard deviation and median with range/interquartile range for continuous variables. Two sample t-test for continuous data and Fisher’s exact test for categorical data were used to assess the difference. To evaluate how itch scores changed over time after retinoid treatment, a linear mixed-effects model was employed. The model for itch scores included time (t), number of retinoids used, baseline itch score, sex, race, and age when retinoid started as fixed effects, whereas a random intercept for subjects was included to account for within-subject correlations. A value of p less than 0.05 was considered statistically significant. Statistics were generated using R version 3.5.3. This study was approved for exemption under the application 2021/00785 from the National Healthcare Group Domain Specific Review Board.

## Results

### High-definition optical coherence tomography demonstrates sweat duct dilation and acrosyringial wall thickening after exposure to heat and sweating in subjects with CPUO

**Figure 1:**
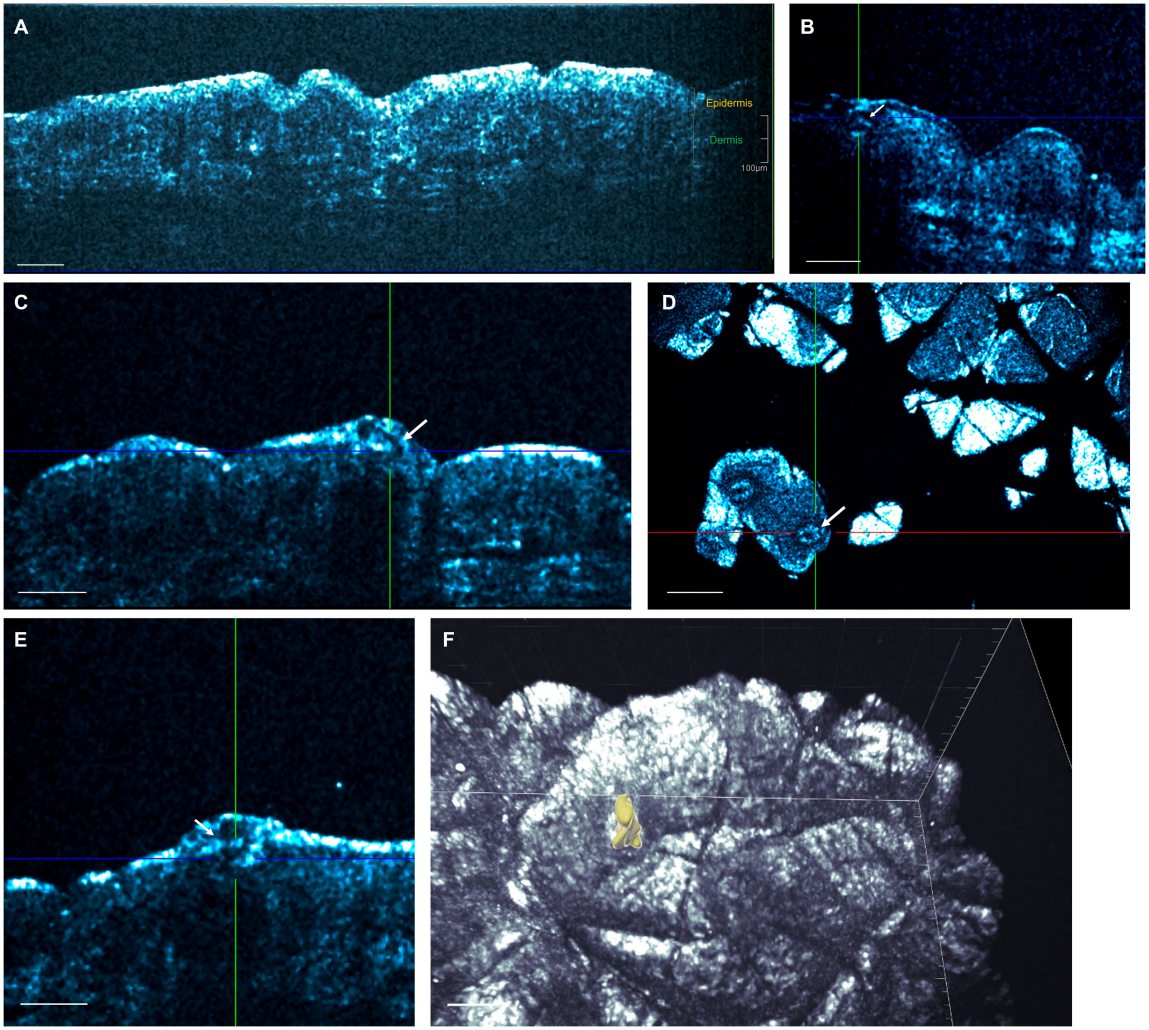
High-definition optical coherence tomography (HD-OCT) of sweat ducts. **(A)** *In vivo* HD-OCT slice view image of a healthy individual’s abdominal skin. Sweat ducts are not normally visualized, except in images of the palms and soles. Scale bar; 200 μm. **(B)** A spiraling sweat duct within the epidermis (acrosyringium) in a patient with CPUO at rest. Scale bar; 100 μm. **(C)** Post-exercise slice image revealing a curved tubular structure in the epidermis representing a dilated acrosyringium. Scale bar; 100 μm. **(D)** In the corresponding *en face* image of **(C)**, tubular structures with thickened walls (one in crosshair), with a surrounding hypo-refractive bordering region indicative of fluid collection can be observed. Scale bar; 200 μm. **(E)** These dilated sweat ducts may present as subcorneal fluid collections, and the dilated sweat duct here is seen to extend into the dermis. Scale bar; 100 μm. **(F)** Three-dimensional rendering demonstrating a dilated and tortuous acrosyringium (yellow structure) in the epidermis. Scale bar; 30 μm. A more comprehensive view is available in Supplementary Video S1.

**Figure 2:**
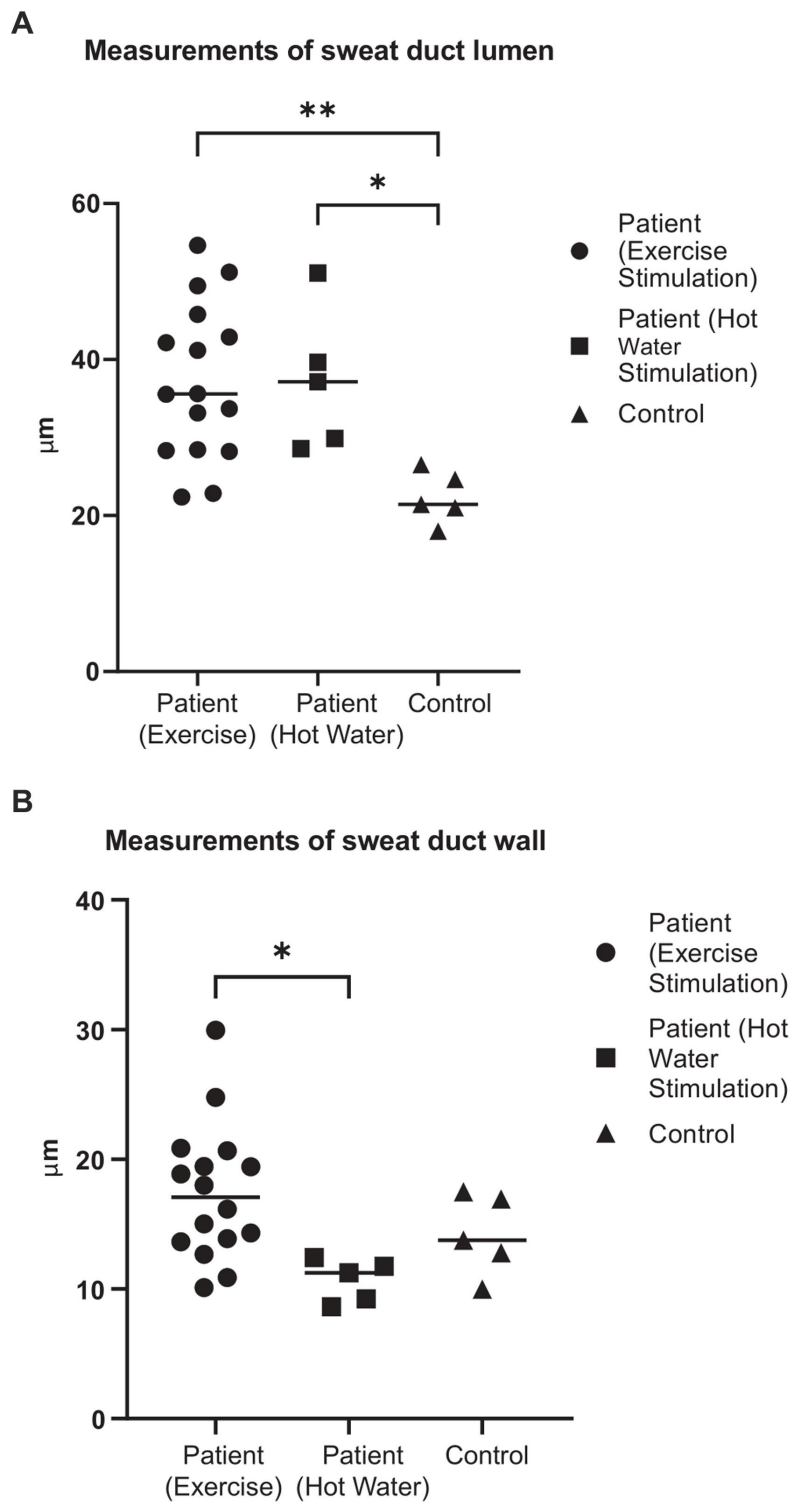
Measurements of sweat duct lumen and wall thickness from HD-OCT images. **(A)** Sweat duct lumen diameter (μm) after induction of heat and sweating in subjects with CPUO and healthy controls. *denotes *p* < 0.05, **denotes *p* < 0.01. **(B)** Sweat duct wall thickness (μm) after induction of heat and sweating in subjects with CPUO and healthy controls. *denotes *p* < 0.05.

In order to visualize irregularities in the sweat duct apparatus, 20 subjects with CPUO underwent HD-OCT skin imaging and heat induction. Fifteen of these patients underwent heat induction by exercise and five, due to being elderly, underwent heat induction through ingestion of hot water in a warm room. HD-OCT identified features of sweat duct obstruction in 19 out of the 20 subjects (Figure 1; Supplementary Video S1), and these features were significantly more prominent post sweat-induction compared to baseline. These abnormalities were not found in healthy individuals. The most prominent features were dilation of the lumen and thickening of the wall of the acrosyringium, which is the spiraling intra-epidermal portion of the sweat duct. This was evidenced by larger sweat duct lumen diameters in affected patients after exercise and hot water stimulation, compared to healthy controls (respective means 37.2 μm and 37.3 μm vs. 22.3 μm, *p* < 0.01 and < 0.05 respectively) (Figure 2A). Concurrently, sweat duct wall thickness was increased in affected patients after exercise stimulation as compared to healthy controls but this did not reach statistical significance (means 17.5 μm vs. 14.7 μm, *p* = 0.21) due to high variability in sweat duct walls (Figure 2B).

The sweat duct dilation was found to form a collection of fluid under the stratum corneum. On the *en face* views, a surrounding hypo-refractive bordering region indicative of fluid accumulation could be observed. The dilation of the sweat ducts and exudation of fluid to the surroundings indicate presence of obstruction of the sweat orifice at the stratum corneum, which is comprised of keratin. We conducted the whole-body starch-iodine sweat testing for the 15 subjects who had exercised and found that they do not have generalized hypohidrosis. We thereby posit that partial, instead of complete, sweat duct obstruction occurs in patients with CPUO.

### Immunostaining demonstrated sweat component accumulation within sweat glands with leakage into the skin

**Figure 3:**
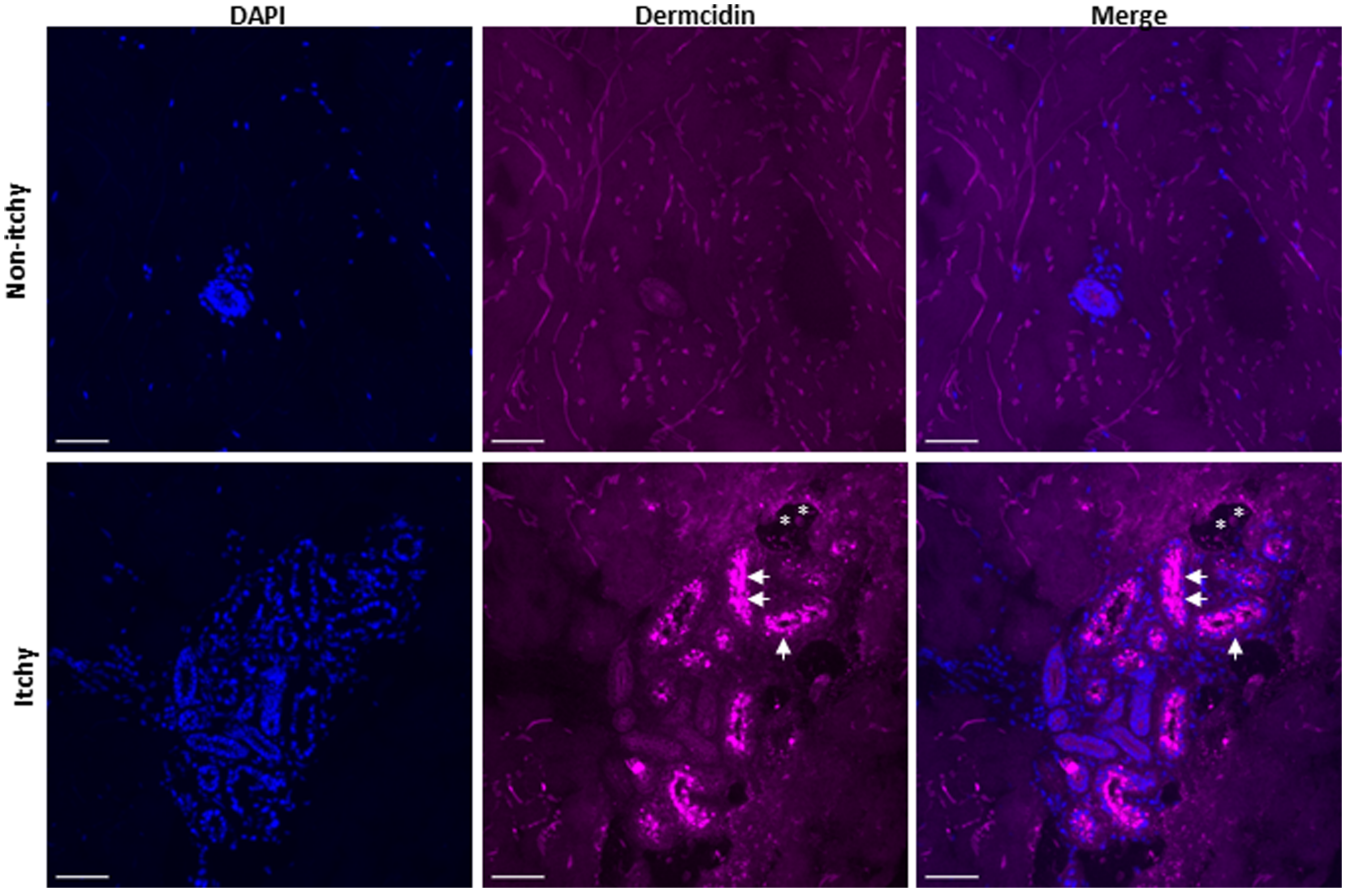
Immunofluorescence staining of sweat glands in a skin biopsy of non-itchy and itchy skin in a patient with CPUO. Scale bar; 50 μm. Images are representative of phenomena in three patients. The skin biopsies were cryosectioned to 10 μm thickness, fixed with paraformaldehyde and immunolabeled for nucleus (DAPI; blue), and dermcidin (magenta). Dermcidin, an antimicrobial peptide present in sweat, can be found accumulated within the secretory sweat glands (arrowheads) with some leakage (asterisk).

In order to further investigate associated pathology occurring in the sweat glands, we carried out immunostaining for dermcidin in skin biopsies of itchy versus non-itchy skin areas in three CPUO patients. Dermcidin is an antimicrobial peptide which is exclusively and constitutively expressed in eccrine sweat glands, and positive staining may be taken as a surrogate for the presence of sweat components (15). Immunostaining demonstrated accumulation of dermicidin in the sweat glands located within the dermis, together with leakage in some cases (Figure 3). Together with the HD-OCT findings above, we theorize that accumulated sweat leaks into the epidermis, irritating intra-epidermal nerve fibers, resulting in itch. Similarly, accumulated sweat which leaks into the dermis could potentially irritate nociceptive/pain nerve fibers, resulting in the prickly sensation that patients often concurrently experience.

Based on these findings indicating keratinaceous partial obstruction of sweat orifices, we treated patients with CPUO with oral retinoids, in the attempt to regulate abnormalities in skin surface keratinization (12, 13), followed by evaluating the change in itch severity experienced.

### Analysis of retinoid efficacy and safety

A total of 56 patients were included in the final analysis, and the baseline characteristics of our study population are as outlined in Table 1. Most of our patients were older, male, and Chinese. The mean duration of itch before their first visit at the Itch Clinic was 116.3 months, and mean itch score at presentation was severe at 8.2. The lower limbs were the most common areas affected. To understand associated comorbidities, we also collected data on suicidal ideation and peripheral eosinophilia. Four (7.1%) patients voluntarily verbalized suicidal ideation due to itch. Thirteen (23.2%) patients had peripheral eosinophilia.

**Table 1:** Baseline characteristics.

Characteristics	Value
Age at first visit, years	
Mean (SD)	55.2 (17.5)
Minimum, maximum	16, 84
Age when systemic retinoid first started, years	
Mean (SD)	58.0 (18.2)
Minimum, maximum	19, 87
Sex	
Male, *n* (%)	39 (69.6)
Female, *n* (%)	17 (30.4)
Race	
Chinese, *n* (%)	48 (85.7)
Malay, *n* (%)	2 (3.6)
Indian, *n* (%)	6 (10.7)
Duration of itch before first visit, months	
Mean (SD)	116.3 (140.4)
Median (Q1, Q3)	48 (24, 180)
Minimum, maximum	1, 528
Baseline itch scores	
Mean (SD)	8.2 (1.8)
Minimum, maximum	3, 10
Areas affected, *n* (%)	
Face and scalp	32 (57.1)
Trunk	46 (82.1)
Upper limbs	49 (87.5)
Lower limbs	51 (91.1)
Verbalized suicidal ideation due to itch, *n* (%)	4 (7.1)
Eosinophilia, *n* (%)	13 (23.2)

SD, standard deviation.

**Figure 4:**
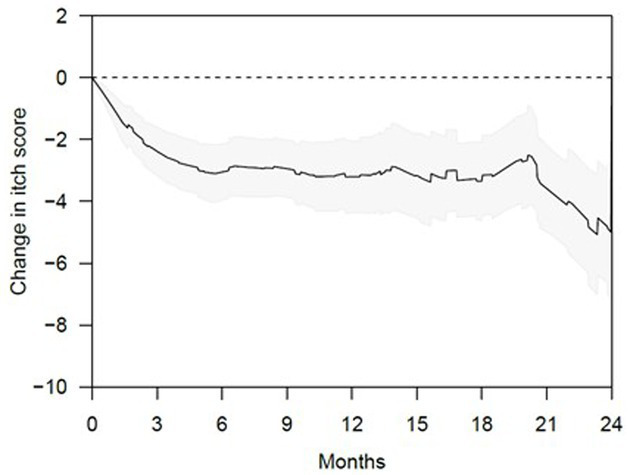
Change in itch scores after systemic retinoid therapy (graphical representation).

Nine patients were treated only with acitretin, 19 only with isotretinoin, and 28 were treated with both retinoids over the course of their follow-up (Table 2). The mean (*SD*) duration of retinoid treatment was 671.9 (397.6) days. Figure 4 and Supplementary Table S1 show the changes in itch scores after starting retinoids. There was a significant reduction in itch score over time, noticeable at 3 months of treatment, during which there was a mean reduction in itch score of 2.38 points (*p* < 0.0001), with the effect being sustained throughout treatment. Thirty-eight patients (67.9%) responded to treatment, defined as having an itch score two points or more lower than baseline, sustained during treatment. A total of eight patients (14.8%) achieved an itch score of 0 or 1 (Supplementary Table S2). Of these, four patients had stopped treatment for a mean of 318.5 days with sustained resolution of itch. Eight patients (14.3%), three on acitretin (8.11%) and five on isotretinoin (10.6%), stopped or changed retinoids due to adverse effects, the most common being dryness (Table 2).

**Table 2:** Treatment data.

Statistics	Value
Patients initially given acitretin, *n* (%)	15 (26.8)
Patients initially given isotretinoin, *n* (%)	41 (73.2)
Patients given acitretin during the course of treatment, *n* (%)	37 (66.1)
Patients given isotretinoin during the course of treatment, *n* (%)	47 (83.9)
Patients who responded to retinoids, *n* (%)	38 (67.9)
**Patients who switched retinoids due to inefficacy, *n* (%)**	**26 (48.2)** ^†^
Eventually responded to retinoids, *n* (%)	13 (50.0)
**Acitretin to isotretinoin, *n* (%)**	**3 (5.36)**
Responded to isotretinoin, *n* (%)	1 (33.3)^‡^
**Isotretinoin to acitretin, *n* (%)**	**19 (33.9)**
Responded to acitretin, *n* (%)	10 (52.6)^§^
**Acitretin to isotretinoin and back to acitretin, *n* (%)**	**2 (3.57)**
Responded to third retinoid, *n* (%)	1 (50.0)
**Isotretinoin to acitretin and back to isotretinoin, *n* (%)**	**1 (1.79)**
Responded to third retinoid, *n* (%)	0 (0.00)
**Isotretinoin to acitretin to isotretinoin to acitretin, *n* (%)**	**1 (1.79)**
Responded to fourth retinoid, *n* (%)	1 (100)
**Patients who stopped or changed retinoids due to side effects, *n* (%)**	**8 (14.3)**
**Acitretin, *n* (%)**	**3 (8.11)**
Stopped retinoid therapy, *n* (%)	2 (66.7)
Changed to isotretinoin, *n* (%)	1 (33.3)
*Side effects experienced*	
Dryness, *n* (%)	2 (66.7)
Worsening of itch, *n* (%)	1 (33.3)
**Isotretinoin, *n* (%)**	**5 (10.6)**
Stopped retinoid therapy, *n* (%)	2 (40.0)
Changed to acitretin, *n* (%)	3 (60.0)
*Side effects experienced*	
Dryness, *n* (%)	3 (60.0)
Abdominal pain, *n* (%)	1 (20.0)
Mood swings, *n* (%)	1 (20.0)

^†^Includes four patients who also switched retinoids due to side effects.

^‡^Of the three patients who switched from acitretin to isotretinoin due to inefficacy, one defaulted follow-up and another did not respond.

^§^Of the 19 patients who switched from isotretinoin to acitretin due to inefficacy, one stopped treatment because of xerosis and eight have not returned for follow-up since.

The values on the indented lines following each bolded line are subsets of the bolded lines, and expressed as percentages of the bolded lines.

## Discussion/conclusion

Management of CPUO remains challenging, and at present, there exists no consistently effective treatment in the literature. Our results indicate that one cause of CPUO is partial sweat duct obstruction, likely by keratinaceous deposits at the skin surface, with corresponding sweat accumulation and leakage into the dermis, and this is evident through the dilatation of the sweat duct upon heat induction observed through *in vivo* HD-OCT imaging, coupled with accumulation and leakage of sweat components into the dermis in immunostaining studies. Correspondingly, in our retrospective cohort, treatment of CPUO patients with retinoids to reduce hyperkeratinization, which could be linked to sweat duct obstruction led to a mean reduction in itch score of 2.38 points (*p* < 0.0001) at 3 months after starting treatment, with sustained effect throughout treatment. Thirty-eight patients (67.9%) responded to treatment, while eight patients (14.3%) achieved no or almost no itch. The results suggest that retinoids can be an effective therapeutic option for CPUO who have failed conventional treatment. By the end of the analysis period, four (7.1%) patients had exhibited a durable disease-free period after stopping treatment, suggesting that the systemic retinoids play a role in address the underlying pathology, rather than providing temporary control such as that conferred by immunosuppressant agents. Moving forward, we should assess for the presence of sweat duct pathology in patients with CPUO and consider the use of retinoids if detected. A low retinoid treatment dose, such as 10 mg daily, appears appropriate. Other clinical implications of the role of sweat gland dysfunction in CPUO would be the need for patient education to maintain a cool environment, wear loose fitting garments and avoid drugs known to induce hypohidrosis.

Isotretinoin and acitretin appear similar in terms of side effect profile. Of note, pregnancy is contraindicated for 3 years after taking acitretin, limiting its use in females of childbearing age, which made up a minority of the patients (8.93%) in our study population (16). The use of isotretinoin would be preferred in this subpopulation.

In our patient cohort with recalcitrant CPUO disease, 32.1% did not respond to either acitretin or isotretinoin. One reason may be the need to address existing secondary inflammation, in addition to the underlying cause. We postulate that the leakage of sweat into the skin, resulting from partial sweat duct obstruction, may trigger the innate immune cells, which include mast cells, basophils, and eosinophils. In our study, we found that 23.2% of our CPUO patients had blood eosinophilia. This is in keeping with a recent retrospective cross-sectional study of patients with chronic pruritus, in which eosinophilia was present in 17.9% of patients with normal-appearing skin and 39.7% of patients presenting with severe chronic secondary scratch lesions (17). Eosinophils are involved in host defense against exogenous antigens and pathogens, and their role in stimulating nerve cells and contributing to pruritus has been demonstrated in several studies (18). Subsequent to triggering of the innate immune system, activation of the adaptive immune system is expected to occur, with the primary involvement of the T-helper 2 pathway. Thus far, immuno-modulatory agents involving antagonism of the interleukins-4, −13 and − 31 and the Janus kinase-signal transducer and activator of transcription pathway have been shown to be useful for controlling itch in various studies (19, 20). However, itch can recur after stopping such treatment because the underlying cause of itch was not addressed. Addition of such immune-modulatory agents to the treatment of underlying structural abnormalities in keratinization may help further optimize treatment outcomes.

CPUO is a disease with significant psychosocial burden. In our study population, 7.1% of patients voluntarily verbalized suicidal ideation due to itch. Expression of suicidal ideation has been associated with a four-fold increased risk for subsequent completed suicides, highlighting the importance of managing pruritus in this group of patients (21).

The limitations of our study are the lack of placebo control due to its retrospective nature, subjective reporting of itch score by patients and the high proportion of patients (60.7%) who remained on systemic retinoids at the conclusion of the study period. A prolonged period of follow-up, especially after cessation of retinoids, will be necessary to assess the proportion of patients with a durable disease-free period. We have only evaluated the use of acitretin and isotretinoin in this study, and other systemic retinoids have not been assessed.

In conclusion, based on the novel findings from physiological imaging studies identifying partial keratinaceous sweat duct obstruction in CPUO, we instituted systemic retinoid treatment to address the underlying pathology. In patients who failed conventional therapies, treatment with isotretinoin or acitretin appears to be an effective and safe therapeutic option.

## Data availability statement

The raw data supporting the conclusions of this article will be made available by the authors, without undue reservation.

## Ethics statement

The requirement of ethical approval was waived by the National Healthcare Group Domain Specific Review Board for the studies involving humans because the study posed no more than minimal risk to research subjects. The studies were conducted in accordance with the local legislation and institutional requirements. The ethics committee/institutional review board also waived the requirement of written informed consent for participation from the participants or the participants’ legal guardians/next of kin because none of the information collected affected the clinical decisions about the individual’s care, and patients were not being deprived of clinical care to which they would normally be entitled.

## Author contributions

SL: Data curation, Formal analysis, Writing – original draft. YT: Conceptualization, Data curation, Formal analysis, Investigation, Methodology, Writing – review & editing. YZ: Data curation, Formal analysis, Writing – review & editing. XZ: Data curation, Formal analysis, Writing – review & editing. LN: Conceptualization, Project administration, Writing – review & editing. HT: Conceptualization, Data curation, Formal analysis, Investigation, Methodology, Project administration, Resources, Supervision, Visualization, Writing – review & editing.

## References

[1] Weisshaar E Dalgard F. Epidemiology of itch: adding to the burden of skin morbidity. Acta Derm Venereol. (2009) 89:339–50. doi: 10.2340/00015555-0662, PMID: 19688144

[2] Matterne U Apfelbacher CJ Loerbroks A Schwarzer T Buttner M Ofenloch R. Prevalence, correlates and characteristics of chronic pruritus: a population-based cross-sectional study. Acta Derm Venereol. (2011) 91:674–9. doi: 10.2340/00015555-1159, PMID: 21879245

[3] Andrade A Kuah CY Martin-Lopez JE Chua S Shpadaruk V Sanclemente G. Interventions for chronic pruritus of unknown origin. Cochrane Database Syst Rev. (2020) 1:CD013128. doi: 10.1002/14651858.CD013128.pub2 31981369 PMC6984650

[4] Chan MMH Lim GH Zhao X Tey HL. Isolated hypohidrosis: pathogenesis and treatment. Eur J Dermatol. (2020) 30:680–7. doi: 10.1684/ejd.2020.3931, PMID: 33262099

[5] Haenssle HA Finkenrath A Hausser I Oji V Traupe H Hennies HC. Effective treatment of severe thermodysregulation by oral retinoids in a patient with recessive congenital lamellar ichthyosis. Clin Exp Dermatol. (2008) 33:578–81. doi: 10.1111/j.1365-2230.2008.02709.x, PMID: 18355358

[6] Sulzberger MB Herrmann F Zak FG. Studies of sweating; preliminary report with particular emphasis of a sweat retention syndrome. J Invest Dermatol. (1947) 9:221–42. doi: 10.1038/jid.1947.92 18918914

[7] Allen HB Vaze ND Choi C Hailu T Tulbert BH Cusack CA. The presence and impact of biofilm-producing staphylococci in atopic dermatitis. JAMA Dermatol. (2014) 150:260–5. doi: 10.1001/jamadermatol.2013.8627, PMID: 24452476

[8] Haque MS Hailu T Pritchett E Cusack CA Allen HB. The oldest new finding in atopic dermatitis: subclinical miliaria as an origin. JAMA Dermatol. (2013) 149:436–8. doi: 10.1001/2013.jamadermatol.109, PMID: 23715198

[9] Silverberg JI Lei D Yousaf M Janmohamed SR Vakharia PP Chopra R. Association of itch triggers with atopic dermatitis severity and course in adults. Ann Allergy Asthma Immunol. (2020) 125:552–559.e2. doi: 10.1016/j.anai.2020.06.014, PMID: 32544530

[10] Lim JH Choo W Chang JH Tey HL Chong WS. Application of iodinated starch powder using an atomizer spray gun – a new and effective tool to evaluate hypohidrosis. Skin Res Technol. (2016) 22:370–4. doi: 10.1111/srt.12275, PMID: 26452436

[11] Lim JH Kok WL Bin Ali N Chong WS Tey HL. Hypohidrosis in individuals with exertional heat injury: a prospective open cohort study. Dermatology. (2016) 232:50–6. doi: 10.1159/000439108, PMID: 26402230

[12] Elias PM. Epidermal effects of retinoids: supramolecular observations and clinical implications. J Am Acad Dermatol. (1986) 15:797–809. doi: 10.1016/S0190-9622(86)70236-3, PMID: 2430000

[13] Torma H. Regulation of keratin expression by retinoids. Dermatoendocrinol. (2011) 3:136–40. doi: 10.4161/derm.15026 22110773 PMC3219164

[14] Reich A Riepe C Anastasiadou Z Medrek K Augustin M Szepietowski JC. Itch assessment with visual analogue scale and numerical rating scale: determination of minimal clinically important difference in chronic itch. Acta Derm Venereol. (2016) 96:978–80. doi: 10.2340/00015555-2433, PMID: 27068455

[15] Schittek B Hipfel R Sauer B Bauer J Kalbacher H Stevanovic S. Dermcidin: a novel human antibiotic peptide secreted by sweat glands. Nat Immunol. (2001) 2:1133–7. doi: 10.1038/ni732, PMID: 11694882

[16] Ormerod AD Campalani E Goodfield MJ Unit BADCS. British Association of Dermatologists guidelines on the efficacy and use of acitretin in dermatology. Br J Dermatol. (2010) 162:952–63. doi: 10.1111/j.1365-2133.2010.09755.x, PMID: 20423353

[17] Lehmann M Cazzaniga S Simon D Perruchoud DL Borradori L Rammlmair A. Patterns among patients with chronic pruritus: a retrospective analysis of 170 patients. Acta Derm Venereol. (2020) 100:adv00068. doi: 10.2340/00015555-3405, PMID: 31950196 PMC9128882

[18] Nakashima C Ishida Y Kitoh A Otsuka A Kabashima K. Interaction of peripheral nerves and mast cells, eosinophils, and basophils in the development of pruritus. Exp Dermatol. (2019) 28:1405–11. doi: 10.1111/exd.14014, PMID: 31365150

[19] Jeon J Wang F Badic A Kim BS. Treatment of patients with chronic pruritus of unknown origin with dupilumab. J Dermatolog Treat. (2021) 33:1754–57. doi: 10.1080/09546634.2021.1880542 33557654

[20] Misery L Brenaut E Pierre O Le Garrec R Gouin O Lebonvallet N. Chronic itch: emerging treatments following new research concepts. Br J Pharmacol. (2021) 178:4775–91. doi: 10.1111/bph.15672, PMID: 34463358

[21] Hubers AAM Moaddine S Peersmann SHM Stijnen T van Duijn E van der Mast RC. Suicidal ideation and subsequent completed suicide in both psychiatric and non-psychiatric populations: a meta-analysis. Epidemiol Psychiatr Sci. (2018) 27:186–98. doi: 10.1017/S2045796016001049, PMID: 27989254 PMC6998965

