# Supplementary material for: In vivo imaging of patients with chronic pruritus of unknown origin reveals partial sweat duct obstruction with partial itch resolution upon retinoid treatment
Source: Front Med (Lausanne). 2023 Sep 22;10:1265148. doi: 10.3389/fmed.2023.1265148 (PMC10556653; doi:10.3389/fmed.2023.1265148)
Supplement: Supplementary file 1 [file Table_1.DOCX]

**Supplementary File for Review**

**Figure S1: Flowchart depicting studies and procedures undergone by subjects in our study**

**Table S1: Change in itch scores after systemic retinoid initiation**

| **Time after starting retinoids, months** | **Number of patients receiving treatment** | **Change in itch score, mean (95% CI)** | **P-value** |
| --- | --- | --- | --- |
| 0 | 56 | 0 (0, 0) | NA |
| 3 | 54 | -2.38 (-3.2, -1.6) | < .0001 |
| 6 | 49 | -3.05 (-4, -2.15) | < .0001 |
| 9 | 47 | -2.94 (-3.9, -2.02) | < .0001 |
| 12 | 43 | -3.21 (-4.4, -2.04) | < .0001 |
| 15 | 38 | -3.17 (-4.6, -1.78) | .0002 |
| 18 | 33 | -3.35 (-4.6, -2.08) | < .0001 |
| 21 | 29 | -3.59 (-5, -2.18) | .0005 |
| 24 | 22 | -5.02 (-7.2, -2.87) | .0059 |

*CI, confidence interval; NA, not applicable*

**Table S2: Characteristics of patients who achieved no or almost no itch (itch score 0 or 1)**

| **Statistics** | **All (n = 8)** |
| --- | --- |
| Starting retinoid dose  Mean, daily dose in mg (SD) | 15.0 (7.1) |
| Maximum retinoid dose  Mean, daily dose in mg (SD) | 16.9 (7.5) |
| Cumulative dose  Mean, dose in mg (SD) | 8347.9 (7334.4) |
| Duration of onset of response  Mean, days (SD) | 141.0 (119.2) |
| Duration of treatment  Mean, days (SD) | 646.4 (375.6) |
| Duration disease-free off treatment^†^  Mean, days (SD) | 318.5 (291.2) |

*SD, standard deviation*

^†^4 patients stopped treatment and had an itch score of 0 or 1 at their latest visit.

**Video S1: Three-dimensional (3D) rendering of dilated acrosyringium in epidermis.**

Manual image segmentation of the lumen of the dilated acrosyringium was carried out on individual image slices to obtain a 3D rendering of the dilated acrosyringium.
