# Supplementary figures and images for: In vivo imaging of patients with chronic pruritus of unknown origin reveals partial sweat duct obstruction with partial itch resolution upon retinoid treatment
Source: Front Med (Lausanne). 2023 Sep 22;10:1265148. doi: 10.3389/fmed.2023.1265148 (PMC10556653; doi:10.3389/fmed.2023.1265148)

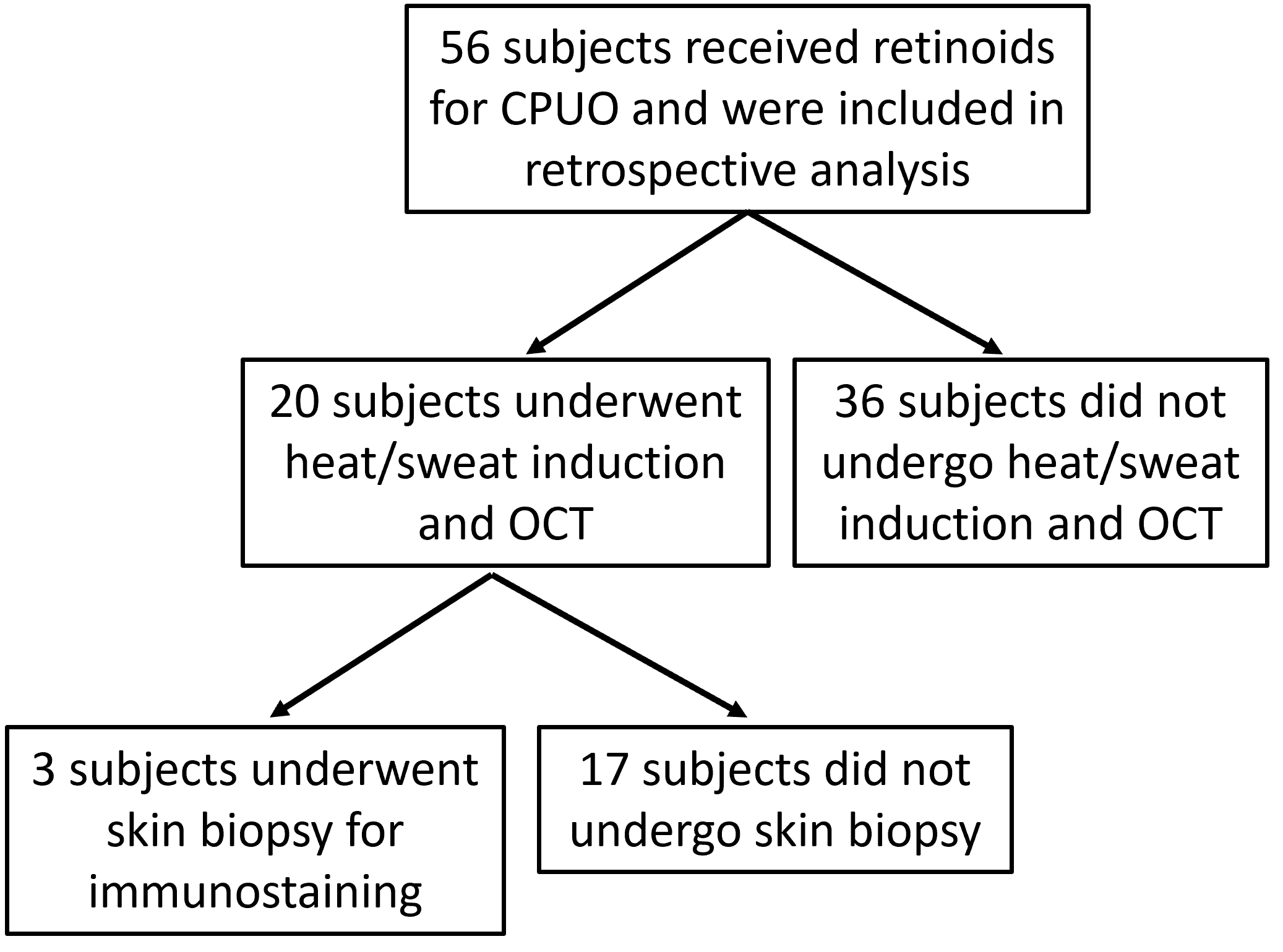

Supplement: Supplementary file 3 [file Image_1.TIF]
